# Targeting Antisense lncRNA PRKAG2-AS1, as a Therapeutic Target, Suppresses Malignant Behaviors of Hepatocellular Carcinoma Cells

**DOI:** 10.3389/fmed.2021.649279

**Published:** 2021-04-13

**Authors:** Yanjiao Ou, Yong Deng, Hong Wang, Qingyi Zhang, Huan Luo, Peng Hu

**Affiliations:** Department of Hepatobiliary Surgery, The First Affiliated Hospital of Army Medical University, Chongqing, China

**Keywords:** PRKAG2-AS1, hepatocellular carcinoma, therapeutic target, proliferation, migration, invasion

## Abstract

**Objective:** Increasing evidence highlights antisense long non-coding RNAs (lncRNAs) as promising therapeutic targets for cancers. Herein, this study focused on the clinical implications and functions of a novel antisense lncRNA PRKAG2-AS1 in hepatocellular carcinoma (HCC).

**Methods:** PRKAG2-AS1 expression was examined in a cohort of 138 HCC patients by RT-qPCR. Overall survival (OS) and disease-free survival (DFS) analyses were presented based on PRKAG2-AS1 expression, followed by ROCs. After silencing PRKAG2-AS1, cell proliferation was assessed via CCK-8, colony formation and EdU staining assays. Migrated and invasive capacities were assessed by wound healing and transwell assays. The relationships between PRKAG2-AS1, miR-502-3p and BICD2 were validated by luciferase reporter, RIP and RNA pull-down assays. The expression and prognostic value of BICD2 were analyzed in TCGA database.

**Results:** PRKAG2-AS1 was up-regulated in HCC than normal tissue specimens. High PRKAG2-AS1 expression was indicative of poorer OS and DFS time. Area under the curves (AUCs) for OS and DFS were 0.8653 and 0.7891, suggesting the well predictive efficacy of PRKAG2-AS1 expression. Targeting PRKAG2-AS1 distinctly inhibited proliferation, migration, and invasion in HCC cells. PRKAG2-AS1 was mainly expressed in cytoplasm of HCC cells. PRKAG2-AS1 may directly bind to the sites of miR-502-3p. Up-regulation of BICD2 was found in HCC tissues and associated with unfavorable prognosis. BICD2 was confirmed to be a downstream target of miR-502-3p. PRKAG2-AS1 could regulate miR-502-3p/BICD2 axis.

**Conclusion:** Our findings identified a novel lncRNA PRKAG2-AS1 that was associated with clinical implications and malignant behaviors. Thus, PRKAG2-AS1 could become a promising therapeutic target.

## Introduction

Hepatocellular carcinoma (HCC) is the third leading cause of cancer-related deaths globally, occupying 75–80% among all liver cancer cases (1). The five-year survival rate is only 15–17% due to recurrence and metastasis (2). Surgical resection followed by transcatheter arterial chemoembolization is the main therapeutic method (3). In recent years, immune and targeted therapies have been widely given wide attention and exhibited favorable clinical outcomes (4, 5). However, only a small percentage of patients will benefit from the latest treatment strategies. Furthermore, molecular target therapy is currently still limited (6). Hence, exploration of novel therapeutic targets is still of significance.

It is well-acknowledged that dysregulated oncogenes or tumor suppressor genes are correlated to carcinogenesis and progression in HCC (7). In the human genome, ~70% is non-coding RNAs (ncRNAs) that are classified into circRNAs, lncRNAs and small ncRNAs according to sequence lengths of transcripts (8). Numerous lncRNAs and circRNAs are indicative to participate in the tumorigenesis and development for HCC (9–11). For instance, an oncogenic antisense lncRNA MCM3AP-AS1 is positively correlated to undesirable clinical outcomes in HCC patients (12). Moreover, its overexpression accelerates tumor malignant growth in HCC. A novel antisense lncRNA PRKAG2-AS1 is overexpressed in colon adenocarcinoma (13), advanced prostate cancer (14) and esophageal squamous cell carcinoma (15). Moreover, its up-regulation could be indicative of unfavorable clinical outcomes of patients. However, the expression and clinical implications of PRKAG2-AS1 remain unclear in HCC. In this study, we identified that this lncRNA was a prognostic factor of HCC patients. Targeting PRKAG2-AS1 could suppress proliferation, migration, and invasion of HCC cells. Hence, it could become a promising therapeutic target.

## Materials and Methods

### Microarray Dataset

From the Gene Expression Omnibus (GEO, http://www.ncbi.nlm.nih.gov/geo) database, microarray data of five pairs of HCC and adjacent normal tissue specimens were obtained in the GSE39678 dataset. Differential expression analysis between HCC and normal tissues was presented via the limma package in R (16). The screening criteria were set as false discovery rate (FDR) < 0.01 and |log2 fold change (FC)| >2.

### Clinical Specimens

Totally, 138 pairs of HCC and adjacent normal tissue specimens were gathered during liver resection at The First Affiliated Hospital of Army Medical University. All patients did not experience any treatment such as transcatheter arterial chemoembolization, immune therapy, or targeted therapy before surgery. Pathological diagnosis was done by two experienced pathologists. Clinical information including age, tumor size, tumor number, HBsAg, tumor differentiation, lymph node metastasis, and clinical stage was obtained for each patient. HCC stage was diagnosed according to the American Joint Committee on Cancer (AJCC) staging standards. All specimens were instantly frozen in liquid nitrogen, followed by being stored at −80°C. Each participant provided the written informed consent. This research met the standards of the Declaration of Helsinki, and was approved by the Ethics Committee of The First Affiliated Hospital of Army Medical University (2018077).

### Cell Culture

Human normal hepatocyte cell line LO2 and HCC cell lines Huh7, HepG2, HCCLM3, and PLC5 were purchased from the Cell Bank of Type Culture Collection of Chinese Academy of Sciences (Shanghai, China). They were grown in DMEM (Gibco, USA) plus 10% FBS in a humidified atmosphere with 5% CO_2_ at 37°C.

### Real-Time Quantitative Polymerase Chain Reaction (RT-qPCR)

Total RNA extraction from tissue specimens or cells was presented utilizing TRIzol reagent (Invitrogen, Carlsbad, California, USA), which was reverse-transcribed into cDNA through the PrimeScript RT reagent kit with gDNA Eraser (Invitrogen). RT-qPCR was performed via the ABI 7500 Fast RT-PCR platform. Using the 2^−ΔΔCt^ method, the relative expression levels of PRKAG2-AS1, miR-502-3p and BICD2 were determined based on the normalization by GAPDH or U6. Table 1 listed the primer sequences.

**Table 1:** The primer sequences utilized in this study for RT-qPCR.

**Names**	**Sequences (5^′^-3^′^)**
PRKAG2-AS1: F	ACTCCAGTTCGAGAAGCCATGC
PRKAG2-AS1: R	CGGAGATCAGCGTTGCAACT
miR-502-3p: F	ACACTCCAGCTGGGAATGCACCTGGGCAAGG
miR-502-3p: R	CTCAACTGGTGTCGTGGA
BICD2: F	CGGAGCGCGAACAGAAGAA
BICD2: R	CAGCATCGTCACTGAACTTGA
GAPDH: F	CGCTCTCTGCTCCTCCTGTTC
GAPDH: R	ATCCGTTGACTCCGACCTTCAC
U6: F	CTCGCTTCGGCAGCACA
U6: R	AACGCTTCACGAATTTGCGT

### Plasmids and Transfection

HCCLM3 and PLC5 cells were transfected by short-hairpin (shRNA) targeting PRKAG2-AS1 (GenePharma, Shanghai, China) as well as non-targeting negative control (NC) plasmids (sh-NC) via lentivectors. The shRNA sequences targeting PRKAG2-AS1 were as follows: sh-1, 5′-ACGAGAATATCATTCAATCTT-3′; sh-2, 5′-TGGTAATGCAGCTTTTCAGTT-3′. miR-502-3p mimics and NC were purchased from Thermo Fisher Scientific (China) Co., Ltd. (Shanghai, China), which were transfected into HCC cells through Lipofectamine 2000 (12566014; Invitrogen, Carlsbad, California, USA) in line with the supplier's specification.

### Cell Counting Kit-8 (CCK-8)

CCK-8 kit (CK04; Dojindo, Japan) was utilized for determination of proliferation of HCCLM3 and PLC5 cells. Briefly, cell suspension was seeded to 96-well plates (100 μL/well). The cells were pre-cultured until the cells grown adherently. Then, 10 μL CCK-8 solution was added to each well. Following being incubated for 2 h, absorbance values at 450 nm were determined with a microplate reader at 48, 72, and 96 h.

### Colony Formation Assay

Forty-Eight hour following transfection, HCCLM3 and PLC5 cell suspension was inoculated in 6-well plates (1 × 10^3^/well) with culture medium lasting 2 weeks. The supernatant was then discarded. Four percentage paraformaldehyde (158127; Sigma, USA) was utilized to fix cell clones lasting 30 min at room temperature. Afterwards, 0.5% crystal violet (Solarbio, Beijing, China) was used to stain the samples lasting 30 min.

### Ethynyl Deoxyuridine (EdU) Incorporation Assay

Cell Proliferation EdU Image Kit (KTA2031; AmyJet Scientific, Wuhan, China) was used for examining the proliferative ability of transfected HCCLM3 and PLC5 cells. Cell suspensions were seeded onto 96-well plates (1 × 10^3^/well). Then, 100 μL 50 μM EdU was added to each well and incubated for 2 h. Cells were fixed by 4% paraformaldehyde (50 μL) for 30 min at room temperature. To neutralize paraformaldehyde, 50 μL 2 mg/mL glycine was added to each well and cultured for 5 min. Afterwards, each well was incubated with 100 μL 0.5% TritonX-100 in PBS for 10 min in order to enhance the permeability of cell membranes. The samples were stained with DAPI dihydrochloride for 5 min. Finally, the images were acquired under a fluorescence microscope (Olympus, Japan).

### Animal Experiment

Twelve BALB/c nude mice aged 4-6 weeks with a body weight of 18–22 g were randomly divided into sh-NC and sh-1 groups. 0.1 mL sh-NC- or sh-1-transfected HCCLM3 cell suspension (1 × 10^8^/mL) was inoculated subcutaneously on the dorsal side of the right hind limb of nude mice. After 28 days, the nude mice were euthanized. The subcutaneous tumor was completely peeled off, and the tumor was weighed. This animal experiment gained the approval of the Animal Ethics Committee of The First Affiliated Hospital of Army Medical University (2018077).

### Wound Healing

Migrated capacity of HCCLM3 and PLC5 cells was assessed via wound healing assay. Firstly, a marker pen was utilized to draw a straight line on the back of 6-well plates. The transfected cells were seeded in the 6-well plates and cultured until confluence was up to 70%. Then, two parallel lines were drawn with a 10 μl pipette tip perpendicular to the marking line. After washing away the scratched cells with PBS, the medium was added to each well. At 0 and 48 h, the images were photographed under an inverted microscope.

### Transwell Assay

Transwell assay was carried out for assessment of invasive capacities of HCCLM3 and PLC5 cells. Briefly, Matrigel (Corning, Shanghai, China) that was diluted with serum-free DMEM was used to coat the upper chambers of transwell insert. 1 × 10^5^ transfected HCCLM3 and PLC5 cells were seeded onto the upper chambers. Moreover, 600 μl DMEM was added to the lower chambers. Following 24 h, 4% paraformaldehyde was used to fix cells. Methanol was added to permeabilize cells. The cells in the upper chambers were then removed. The cells in the lower chambers were stained with 0.3% crystal violet dye. Under a light microscope (×200), the results were acquired and the number of invasive cells were counted.

### Western Blot

Transfected HCCLM3 and PLC5 cells were lysed utilizing RIPA buffer (Beyotime, Shanghai, China). The quality of protein samples was assessed via BCA method. Then, the samples were separated by SDS–PAGE, followed by being transferred to PVDF membranes. The membranes were blocked with 5% skimmed milk powder blocking solution at room temperature for 2 h. Afterwards, the membranes were incubated with primary antibodies against E-cadherin (1:10000; ab40772, Abcam, USA) N-cadherin (1:1000; ab76057, Abcam), vimentin (1:1000; 741, Cell Signaling Technology, USA), BICD2 (1:1000; ab237616, Abcam), and GAPDH (1:1000; ab181602, Abcam) overnight at 4°C, followed by being incubated with HRP-conjugated secondary antibodies (1:1000; ab7097, Abcam) at room temperature lasting 2 h. Enhanced chemiluminescence kit (Beyotime) was used for visualizing the protein bands. The grayscale density was measured by ImageJ software.

### Extraction of Nuclear and Cytoplasmic RNAs

Nuclear and cytoplasmic RNAs were isolated from HCCLM3 and PLC5 cells utilizing the PARIS kit (Invitrogen) in line with the supplier's specification. PRKAG2-AS1 expression was detected by RT-qPCR.

### Luciferase Reporter Assay

Fragments of PRKAG2-AS1 that contained the predicted miR-502-3p binding sites were amplified using PCR, which were used for forming the reporter vector PRKAG2-AS1-wild-type (PRKAG2-AS1-wt). The putative binding sites of miR-502-3p in the PRKAG2-AS1 were mutated, called as PRKAG2-AS1-mutated-type (PRKAG2-AS1-MuT). Meanwhile, 3′-UTR of BICD2 was amplified using PCR and reporter vector BICD2-wt was constructed. BICD2-MuT was then formed. Then, miR-502-3p mimics or inhibitors were transfected into HCCLM3 and PLC5 cells via Lipofectamine 2000 reagent (Invitrogen). Finally, the luciferase activity was measured through the Dual-Luciferase Reporter Assay (GenePharma, Shanghai, China).

### RNA Immunoprecipitation (RIP)

The relationships between PRKAG2-AS1, miR-502-3p and Ago2 were detected using RIP kits (Millipore, USA). The HCCLM3 and PLC5 cells were lysed with RIPA lysis buffer lasting 5 min. After being resuspended with RIP wash buffer, beads were incubated with 5 μg antibodies against Ago2 (1:50, ab156870; Abcam) and IgG (1:100, ab133470; Abcam) for binding. Then, following being resuspended, the magnetic bead-antibody complexes were harvested on a magnetic base. The samples and inputs were digested using proteinase K. Extracted RNA was detected using RT-qPCR.

### RNA Pull-Down

HCCLM3 and PLC5 cells were transfected with 50 nM biotinylated PRKAG2-AS1-wt and PRKAG2-AS1-MuT (GenePharma). Following 48 h, the harvested cells were lysed, which were incubated overnight at 4 °C with beads (Sigma, USA). At last, the bound RNAs were purified through Trizol reagent. The enrichment of miR-502-3p was measured via RT-qPCR.

### Statistical Analysis

One hundred thirty-eight HCC patients were separated into high and low PRKAG2-AS1 expression groups in line with its median value. To evaluate the prognostic value of PRKAG2-AS1, Kaplan-Meier curves were depicted for overall survival (OS) and disease-free survival (DFS). The associations of PRKAG2-AS1 expression with clinicopathological characteristics were determined via Chi-square test. Receiver operating characteristic curves (ROCs) for OS and DFS were presented to assess whether PRKAG2-AS1 expression accurately and sensitively predicted the clinical outcomes. Multivariate analyses were presented to assess the association of PRKAG2-AS1 expression and clinicopathological characteristics with OS and DFS by Cox regression model. Form the Cancer Genome Atlas (TCGA), the expression and clinical implications of BICD2 were analyzed in HCC. *P* < 0.05 was considered statistically significant.

## Results

### Antisense lncRNA PRKAG2-AS1, as a Prognostic Factor, Is Up-Regulated in HCC Tissues and Cells

**Figure 1:**
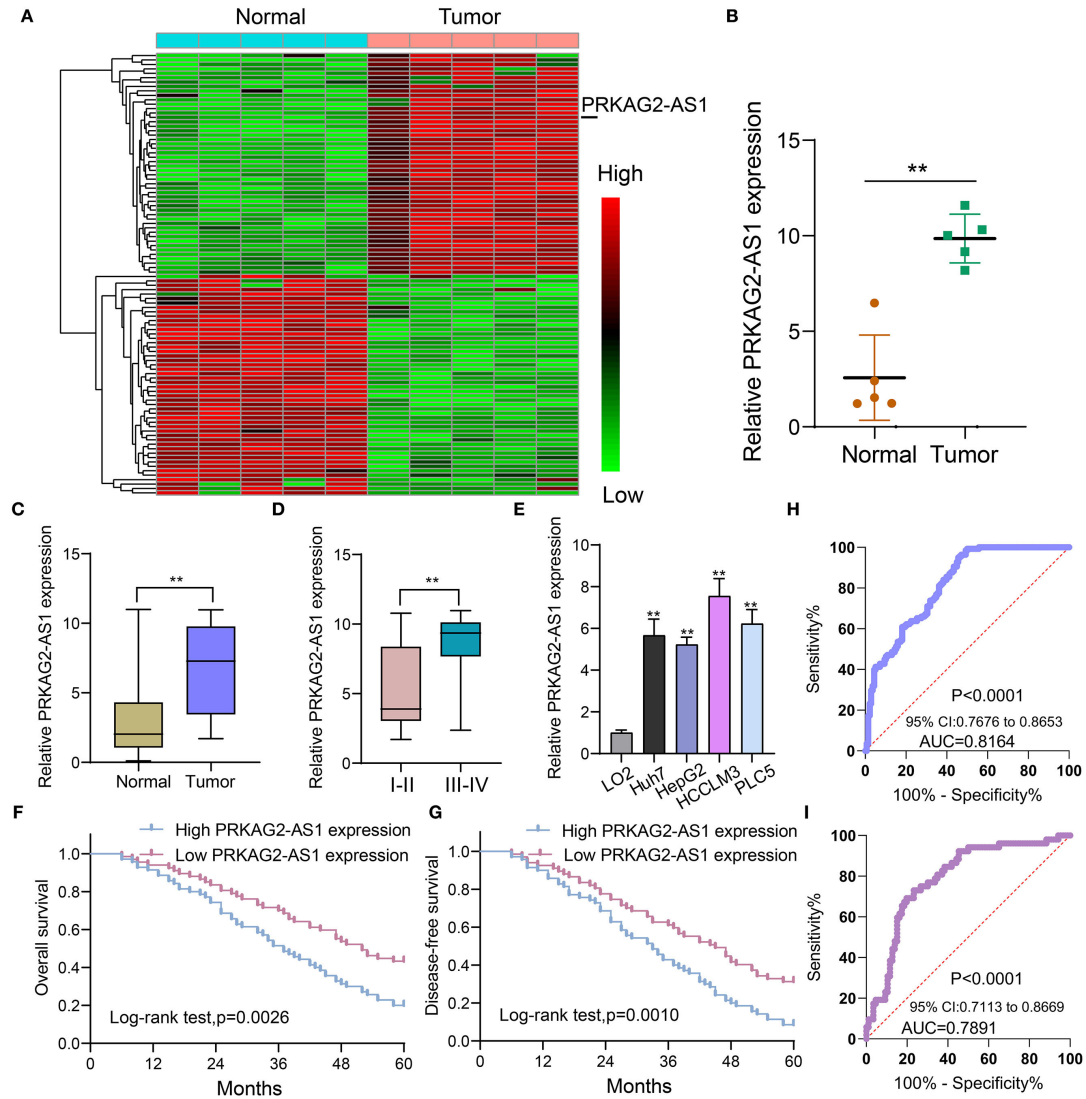
Antisense lncRNA PRKAG2-AS1 is up-regulated both in HCC tissues and cells and could predict clinical outcomes of patients. **(A)** Heat map for dysregulated expressed lncRNAs between HCC and normal tissue specimens from the GEO database. Red: up-regulation and green: down-regulation. **(B)** According to microarray results, PRKAG2-AS1 expression was visualized between HCC and normal tissue specimens. **(C)** RT-qPCR confirming the up-regulation of PRKAG2-AS1 in HCC than normal tissue specimens in an HCC cohort (*n* = 138). **(D)** Violin diagram for the differences in PRKAG2-AS1 expression between stage I-II and III-IV HCC patients. **(E)** RT-qPCR examining PRKAG2-AS1 expression in normal liver cells (LO2) and four kinds of HCC cells (Huh7, HepG2, HCCLM3, and PLC5). **(F,G)** Kaplan-Meier curves for OS and DFS among HCC patients, assessed by log-rank test. **(H,I)** ROCs for OS and DFS based on PRKAG2-AS1 expression. **p < 0.01.

Herein, we analyzed dysregulated lncRNAs between 5 pairs of HCC and normal tissue specimens from the GEO database (Figure 1A). Among all differentially expressed lncRNAs, a novel antisense lncRNA PRKAG2-AS1 caught our attention. It was significantly up-regulated in HCC than normal tissue specimens (Figure 1B). We further expanded the sample size to verify its expression. In a cohort of 138 HCC patients, its up-regulation was detected in HCC than normal tissue specimens, which was consistent with microarray consequences (Figure 1C). Moreover, we compared the differences in its expression between stage I–II and III–IV. As shown in Figure 1D, higher expression of PRKAG2-AS1 was found in stage III-IV than I-II, indicating that PRKAG2-AS1 might be correlated to HCC progression. We also examined PRKAG2-AS1 expression in different kinds of HCC cell lines. Compared to LO2 normal liver cells, its higher expression was detected in Huh7, HepG2, HCCLM3 and PLC5 HCC cell lines (Figure 1E). Association of PRKAG2-AS1 expression with clinicopathological characteristics of HCC patients was further analyzed in our cohort. In Table 2, PRKAG2-AS1 expression was distinctly correlated to lymph node metastasis (*p* = 0.015) and stage (*p* = 0.007). Also, patients with high PRKAG2-AS1 expression usually experienced shorter OS time (*p* = 0.0026; Figure 1F) and DFS time (*p* = 0.0010; Figure 1G). To further verify the predictive performance of PRKAG2-AS1 expression, we conducted ROCs for OS and DFS. The data suggested that the area under the curves (AUCs) for OS and DFS were 0.8164 (Figure 1H) and 0.7891 (Figure 1I), respectively. Multivariate analyses revealed that PRKAG2-AS1 expression could independently predict OS (HR: 2.955; 95%CI: 1.211–4.675; *p* = 0.015) and DFS (HR: 3.118; 95%CI: 1.345–5.122; *p* = 0.009) for HCC patients (Table 3). Hence, PRKAG2-AS1 could be a robust prognostic factor for HCC.

**Table 2:** Association of PRKAG2-AS1 expression with clinicopathological characteristics of HCC patients.

**Parameters**	**Group**	**Total**	**PRKAG2-AS1 expression**		***P*-value**
			**High**	**Low**	
Gender	Male	79	38	41	0.476
	Female	59	32	27	
Age (years)	<60	71	33	38	0.304
	≥60	67	37	30	
Tumor size (cm)	<5	77	36	41	0.294
	≥5	61	34	27	
Tumor number	Solitary	64	31	33	0.617
	Multiple	74	39	35	
HBsAg	Positive	92	47	45	0.904
	Negative	46	23	23	
Tumor differentiation	Well/moderate	85	38	47	0.073
	Poor	53	32	21	
Lymph node metastasis	Absence	94	41	53	0.015
	Presence	44	29	15	
Clinical stage	I-II	86	36	50	0.007
	III-IV	52	34	18	

**Table 3:** Multivariate analyses for overall survival and disease-free survival by Cox regression model.

**Variable**	**Overall survival**			**Disease-free survival**		
	**HR**	**95% CI**	***P***	**HR**	**95% CI**	***P***
Gender	1.453	0.678–1.932	0.323	1.378	0.785–2.221	0.213
Age	1.132	0.564–1.893	0.233	1.324	0.656–2.341	0.159
Tumor size	1.455	0.832–2.438	0.132	1.665	0.986–2.513	0.091
Tumor number	1.376	0.683–1.982	0.345	1.469	0.773–2.158	0.243
HBsAg	0.867	0.472–1.475	0.562	0.943	0.656–1.883	0.676
Tumor differentiation	1.447	0.956–2.218	0.091	1.554	1.038–2.145	0.086
Lymph node metastasis	3.362	1.348-5.672	0.001	3.672	1.554-6.329	0.001
Clinical stage	2.867	1.286-4.879	0.012	3.018	1.345-5.334	0.007
PRKAG2-AS1 expression	2.955	1.211-4.675	0.015	3.118	1.345-5.122	0.009

### Targeting PRKAG2-AS1 Suppresses Proliferative Ability of Hcc Cells

**Figure 2:**
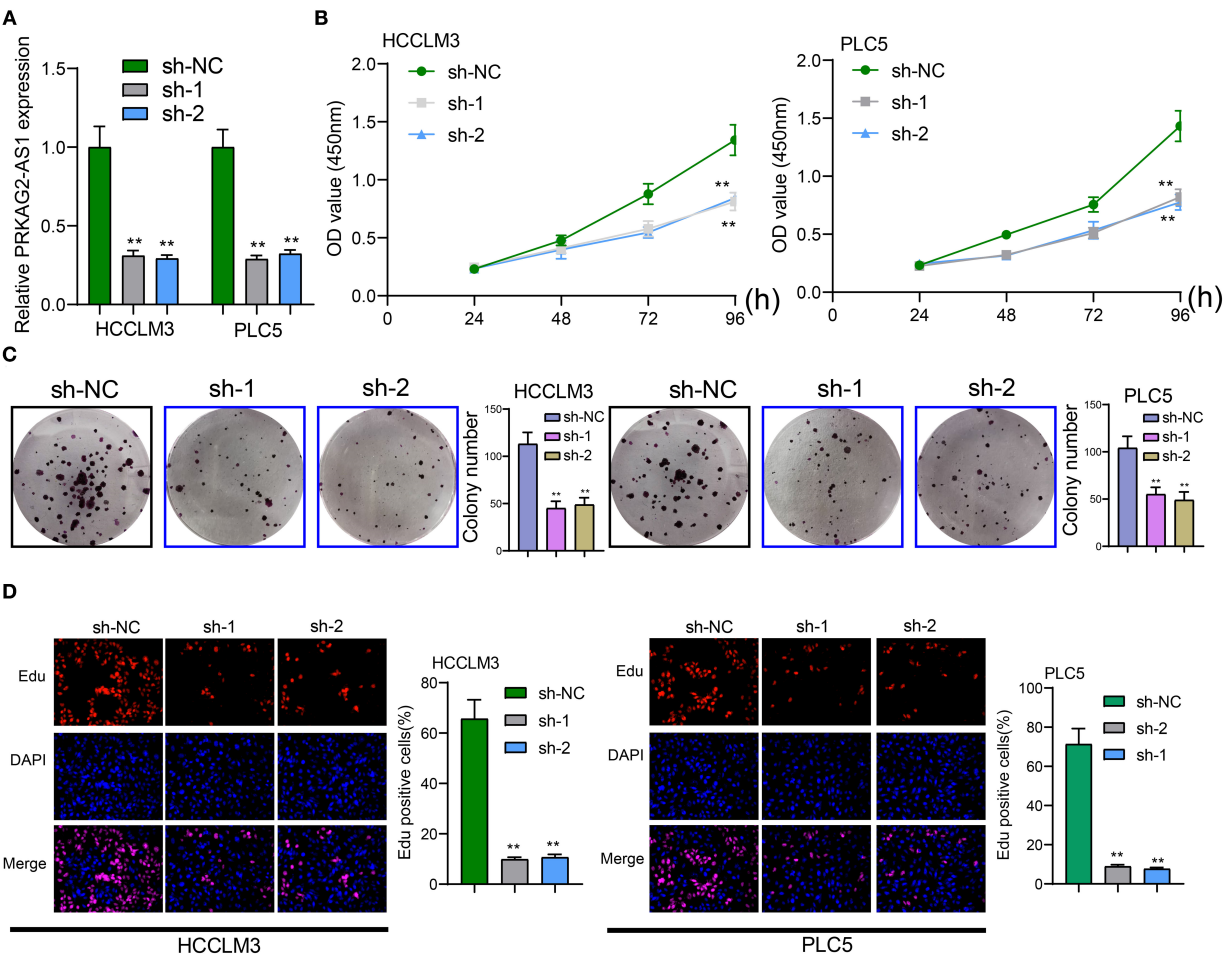
Targeting PRKAG2-AS1 inhibits proliferative capacity of HCC cells. **(A)** RT-qPCR confirmed the successful suppression of PRKAG2-AS1 expression by two shRNAs. **(B)** CCK-8 for examining the relative cell viability of HCC cells after transfection with shRNAs for 48, 72, and 96 h. **(C)** The colony formation was evaluated for HCCLM3 and PLC5 cells with PRKAG2-AS1 knockdown. **(D)** Representative images of EdU staining for HCC cells with PRKAG2-AS1 knockdown. **p < 0.01.

The roles of PRKAG2-AS1 on malignant behaviors of HCC cells were investigated in depth. Two shRNAs targeting PRKAG2-AS1 were transfected into HCCLM3 and PLC5 cells. RT-qPCR confirmed that PRKAG2-AS1 expression was stably silenced (Figure 2A). As shown in CCK-8 results, PRKAG2-AS1 knockdown distinctly slowed down the proliferation of HCCLM3 and PLC5 cells (Figure 2B). Moreover, clone formation assay demonstrated that, compared to sh-NC group, the number of clones of HCC cells was significantly lessened following silencing PRKAG2-AS1 (Figure 2C). EdU staining results also confirmed that the proliferative ability of HCCLM3 and PLC5 cells was markedly restrained by PRKAG2-AS1 knockdown in Figure 2D. Collectively, targeting PRKAG2-AS1 may suppress proliferation of HCC cells.

### Silencing PRKAG2-AS1 Suppresses Tumor Growth

**Figure 3:**
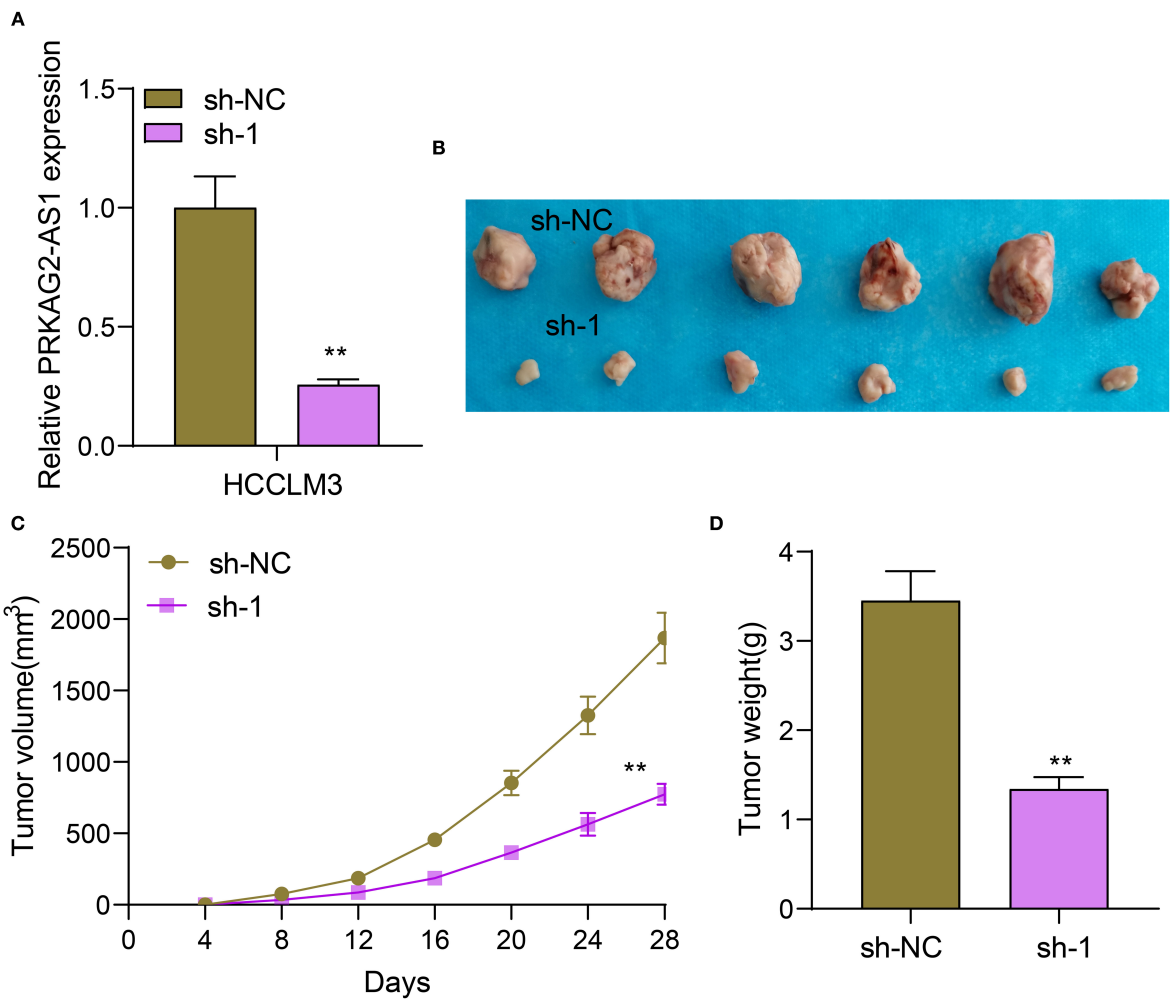
Silencing PRKAG2-AS1 suppresses tumor growth. **(A)** RT-qPCR for the expression of PRKAG2-AS1 in HCCLM3 cells transfected with sh-NC and sh-1 targeting PRKAG2-AS1. **(B)** Tumor images from nude mice inoculated with HCCLM3 cells transfected with sh-NC and sh-1. **(C)** The tumor growth curves. **(D)** Measurement of the tumor weights. **p < 0.01.

Nude mouse tumorigenicity assay was presented by inoculating HCCLM3 cells transfected with sh-NC or sh-1 targeting PRKAG2-AS1 (Figure 3A). After 28 days, we removed the tumors of two groups. In Figure 3B, tumor volume was distinctly decreased in sh-1 group than sh-NC group. The growth curves of tumor volume were depicted, as shown in Figure 3C. The tumor growth was distinctly suppressed by sh-1 targeting PRKAG2-AS1. We also measured the tumor weights of tumors in two groups. Lower tumor weights were detected after silencing PRKAG2-AS1 (Figure 3D).

### Targeting PRKAG2-AS1 Inhibits Migration and Invasion of HCC Cells

**Figure 4:**
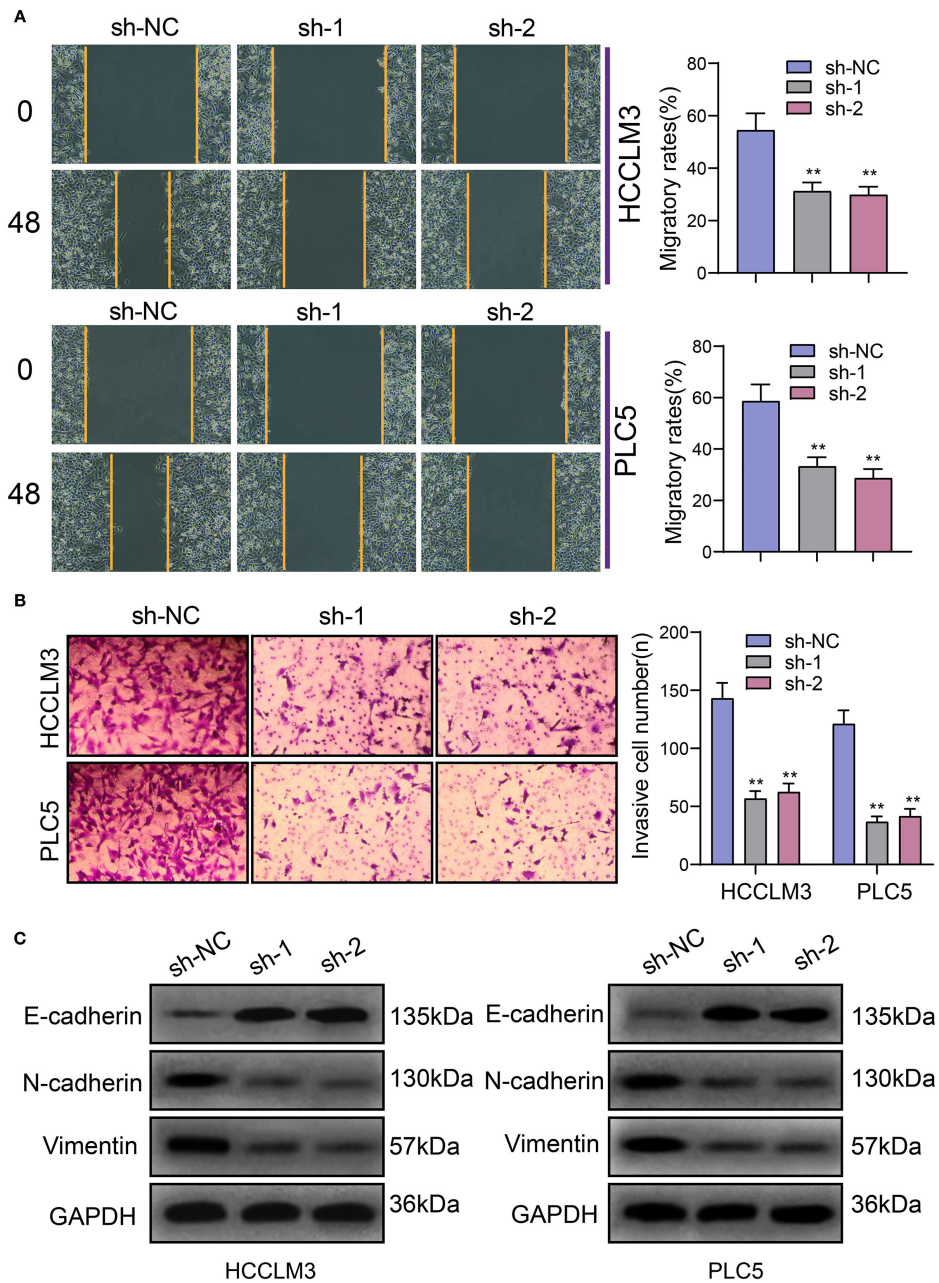
Targeting PRKAG2-AS1 inhibits migration, invasion, and EMT process of HCC cells. **(A)** Wound healing assay for assessment of the migrated capacity of HCCLM3 and PLC5 cells transfected with PRKAG2-AS1 shRNAs. **(B)** Transwell assay for examining the number of invasive cells after PRKAG2-AS1 knockdown. **(C)** Western blot for measuring the expression of EMT-related proteins including E-cadherin, N-cadherin, and Vimentin in HCC cells transfected with PRKAG2-AS1 shRNAs or sh-NC. **p < 0.01.

The migrated and invasive capacities of HCC were further assessed following transfection with PRKAG2-AS1 shRNAs. In Figure 4A, compared to sh-NC group, wound distance was significantly wider in HCCLM3 and PLC5 cells with PRKAG2-AS1 knockdown. Furthermore, transwell assay was presented to evaluate the invasive ability of transfected HCC cells. The data showed that the number of invasive cells was distinctly decreased following transfection with PRKAG2-AS1 shRNAs (Figure 4B). Western blot was used for evaluation of the expression of epithelial-to-mesenchymal transition (EMT)-related proteins in HCC cells. In Figure 4C, E-cadherin expression was markedly increased in HCCLM3 and PLC5 cells with PRKAG2-AS1 knockdown. Meanwhile. N-cadherin and Vimentin expression was both decreased in the two HCC cells transfected with PRKAG2-AS1 shRNAs. Taken together, targeting PRKAG2-AS1 inhibited migration, invasion and EMT process of HCC cells.

### LncRNA PRKAG2-AS1 Can Bind to miR-502-3p

**Figure 5:**
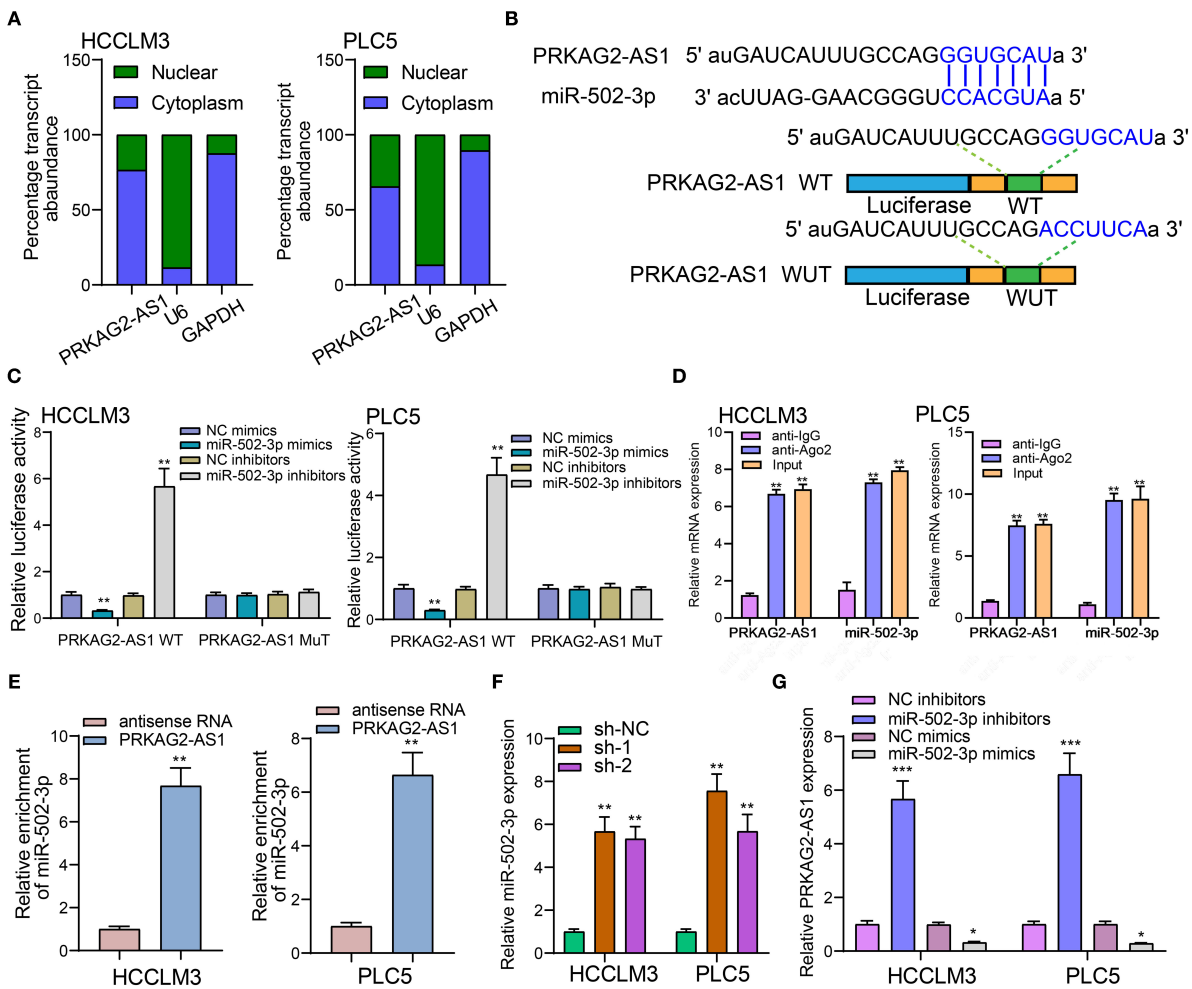
LncRNA PRKAG2-AS1 can bind to miR-502-3p. **(A)** The cellular distribution of PRKAG2-AS1 expression. U6 as a control of nuclear and GAPDH as a control of cytoplasm. **(B)** The schematic diagram of the binding sites between PRKAG2-AS1 and miR-502-3p. **(C)** The luciferase reporter assay results confirming the direct binding relationship between PRKAG2-AS1 and miR-502-3p. **(D)** RIP results for the binding of PRKAG2-AS1 to Ago2. **(E)** RNA pull-down for the enrichment levels of miR-502-3p in HCC cells transfected with PRKAG2-AS1-WT and PRKAG2-AS1-MuT. **(F)** RT-qPCR for detection of miR-502-3p expression in HCC cells with PRKAG2-AS1 knockdown. **(G)** RT-qPCR examining PRKAG2-AS1 expression in HCC cells transfected with miR-502-3p mimics or inhibitors. *p < 0.05; **p < 0.01; ***p < 0.001.

We firstly detected the cellular distribution of PRKAG2-AS1 in HCCLM3 and PLC5 cells. In Figure 5A, PRKAG2-AS1 was mainly expressed in cytoplasm. Based on the DIANA-LncBase v2 database (http://www.microrna.gr/LncBase) (17), there was a binding region between PRKAG2-AS1 and miR-502-3p (Figure 5B). Luciferase reporter assay results confirmed that PRKAG2-AS1 directly bound to the predicted region of miR-502-3p. As shown in Figure 5C, when transfection with miR-502-3p mimics, the relative luciferase activity was distinctly decreased in HCC cells transfected with PRKAG2-AS1-WT not PRKAG2-AS1-MuT. The RIP results demonstrated the binding of PRKAG2-AS1 to Ago2 (Figure 5D). Moreover, RNA pull-down confirmed that more miR-502-3p was enriched in HCC cells with RKAG2-AS1-WT than those with PRKAG2-AS1-MuT (Figure 5E). After silencing PRKAG2-AS1, miR-502-3p expression was markedly decreased in two HCC cells (Figure 5F). Additionally, in HCC cells transfected with miR-502-3p inhibitors, PRKAG2-AS1 expression was distinctly elevated (Figure 5G). On the contrary, its expression was significantly reduced when transfection with miR-502-3p mimics. These findings revealed that PRKAG2-AS1 can bind to miR-502-3p.

### BICD2, as a Prognostic Factor for HCC, Is a Potential Target of miR-502-3p

**Figure 6:**
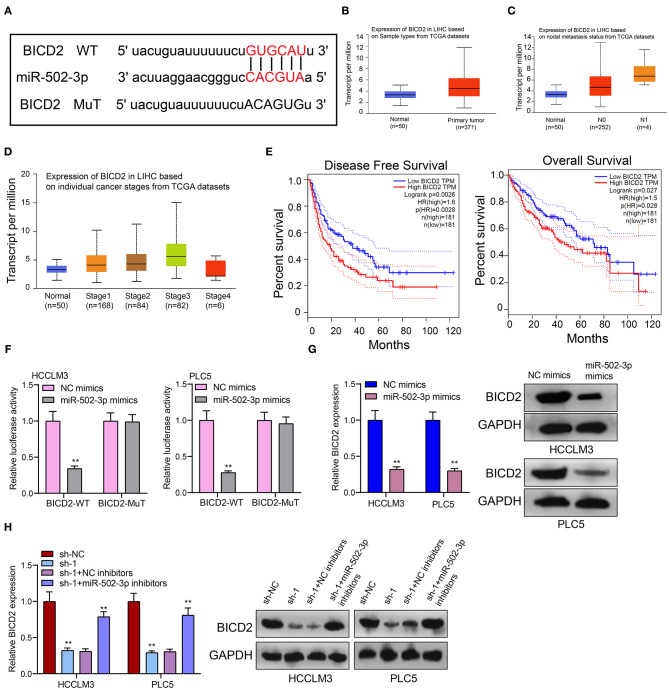
BICD2 is a prognostic factor for HCC and is a potential target of miR-502-3p. **(A)** The schematic diagram of the binding sites between BICD2 and miR-502-3p. **(B)** Box plots for the expression of BICD2 in HCC and normal samples from TCGA database. **(C)** The differences in BICD2 expression in HCC patients with or without nodal metastasis. **(D)** The expression of BICD2 among different stages. **(E)** Kaplan-Meier curves for DFS and OS between high and low BICD2 expression among HCC patients. **(F)** The luciferase reporter assay for the direct relationship between BICD2 and miR-502-3p. **(G)** Western blot for BICD2 expression in HCC cells with miR-502-3p mimics or NC. **(H)** Western blot for BICD2 expression in HCC cells with PRKAG2-AS1 shRNA and/or miR-502-3p inhibitors. **p < 0.01.

From the starbase database (http://starbase.sysu.edu.cn/) (18), there was a binding site between miR-502-3p and BICD2 (Figure 6A), indicating that BICD2 could be a potential target of miR-502-3p. In TCGA database, we analyzed the clinical implications of BICD2 expression in HCC. In Figure 6B, BICD2 was significantly up-regulated in HCC than normal tissue specimens. Moreover, patients with nodal metastasis showed higher BICD2 expression compared to those without nodal metastasis (Figure 6C). As depicted in Figure 6D, there was a distinct difference in BICD2 expression among different stages. Patients with high BICD2 expression was indicative of poorer DFS (*p* = 0.0026) and OS (*p* = 0.027) in comparison to those with its low expression (Figure 6E). These data demonstrated that BICD2 was an underlying prognostic marker for HCC. The luciferase reporter assay results confirmed the direct relationship between BICD2 and miR-502-3p (Figure 6F). miR-502-3p mimics markedly reduced BICD2 expression in HCCLM3 and PLC5 cells (Figure 6G). Furthermore, PRKAG2-AS1 knockdown distinctly lowered BICD2 expression (Figure 6H). However, miR-502-3p inhibitors ameliorated the decrease in BICD2 expression in HCC cells. Thus, PRKAG2-AS1 could mediate miR-502-3p/BICD2 axis in HCC.

## Discussion

HCC is a commonly diagnosed malignancy of the digestive system (19–21). Its pathogenesis involves in complex multiple factors and steps (22). There is still no curative treatment targeting HCC (23). Hence, it is of clinical significance to probe promising therapeutic targets. Herein, this study identified a novel antisense lncRNA PRKAG2-AS1, which could robustly predict unfavorable prognosis. Targeting PRKAG2-AS1 could suppress malignant behaviors. In consequence, this novel could be a prospective therapeutic target for HCC.

Up-regulation of PRKAG2-AS1 was detected in HCC than normal tissue specimens. As previous studies, this lncRNA could be overexpressed in colon adenocarcinoma (13), advanced prostate cancer (14) and esophageal squamous cell carcinoma (15). Our data suggested that PRKAG2-AS1 up-regulation was suggestive of poor OS and DFS. As confirmed by ROCs, PRKAG2-AS1 expression accurately and sensitively predicted clinical outcomes of HCC patients. Also, its expression was markedly correlated to lymph node metastasis and stage. As validated by multivariate analyses, PRKAG2-AS1 expression could independently predict OS and DFS. Thus, PRKAG2-AS1 could be a promising prognostic factor for HCC. Previously, PRKAG2-AS1 has been identified as a prognostic marker for colon adenocarcinoma (13), advanced prostate cancer (14) and esophageal squamous cell carcinoma (15).

Our further analyses revealed that targeting PRKAG2-AS1 distinctly suppressed malignant biological behaviors including proliferation, migration, as well as invasion. Epithelial-to-mesenchymal transition (EMT) is involved in invasion and metastasis in various cancers (24). Numerous studies have verified its key role in HCC development. For example, Wang et al. found that lncRNA CASC2 may suppress EMT process of HCC cells via CASC2/miR-367/FBXW7 axis (25). Our data suggested that targeting PRKAG2-AS1 elevated E-cadherin expression, while lowered N-cadherin and Vimentin expression in HCC cells, thereby inactivating EMT process. Numerous up-regulated lncRNAs have been found to facilitate malignant biological behaviors, such as lncRNA-BC200 (26), lncRNA FAL1 (27), lncRNA SNHG7 (28), and the like.

PRKAG2-AS1 was mainly distributed in the cytoplasm of HCC cells. After validation by luciferase reporter, RIP and RNA pull-down assays, PRKAG2-AS1 can directly bind to miR-502-3p. There were mutual regulatory relationships between the two. miR-502-3p down-regulation has been found in various cancers. For example, its down-regulation could facilitate proliferative and migrated capacities of gallbladder cancer cells (29). Low miR-502-3p expression accelerates proliferation as well as migration for gastric cancer cells via NRAS/MEK1/ERK1/2 axis (30). Here, our study identified that PRKAG2-AS1 was a sponge of miR-502-3p, thereby promoting HCC progression. By verification of luciferase reporter assay, BICD2 was a downstream target of miR-502-3p. It was up-regulated in HCC than normal tissues and was associated with nodal metastasis, stage, and clinical outcomes. Hence, BICD2 could be a promising prognostic marker for HCC. BICD2 suppressed by miR-340 is up-regulated in pancreatic cancer (31). It can also enhance vinorelbine-mediated mitotic arrest in non-small-cell lung cancer cells (32). miR-502-3p mimics could lower BICD2 expression. Furthermore, miR-502-3p inhibitors ameliorated the decrease in BICD2 expression induced by PRKAG2-AS1 knockdown. Therefore, PRKAG2-AS1 could mediate miR-502-3p / BICD2 axis in HCC.

## Conclusion

Collectively, our study identified a novel antisense up-regulated lncRNA PRKAG2-AS1 for HCC. The up-regulation independently predicted worse clinical outcomes of patients. Targeting PRKAG2-AS1 may restrain malignant behaviors. In terms of mechanism, PRKAG2-AS1 could mediate miR-502-3p / BICD2 axis, thereby facilitating the progression of HCC. Hence, PRKAG2-AS1 could be a possible therapeutic target for HCC.

## Data Availability Statement

The datasets presented in this study can be found in online repositories. The names of the repository/repositories and accession number(s) can be found in the article/Supplementary Material.

## Ethics Statement

The studies involving human participants were reviewed and approved by The Ethics Committee of The First Affiliated Hospital of Army Medical University (2018077). The patients/participants provided their written informed consent to participate in this study.

## Author Contributions

PH conceived and designed the study. YO and YD conducted most of the experiments and data analysis, and wrote the manuscript. HW, QZ, and HL participated in collecting data and helped to draft the manuscript. All authors reviewed and approved the manuscript.

## Conflict of Interest

The authors declare that the research was conducted in the absence of any commercial or financial relationships that could be construed as a potential conflict of interest.

## Abbreviations

lncRNAs: long non-coding RNAs
HCC: hepatocellular carcinoma
OS: overall survival
DFS: disease-free survival
ncRNAs: non-coding RNAs
GEO: gene expression omnibus
FDR: false discovery rate
FC: fold change
RT-qPCR: real-time quantitative polymerase chain reaction
NC: negative control
CCK-8: cell counting kit-8
EdU: ethynyl deoxyuridine
RIP: RNA immunoprecipitation
ROCs: receiver operating characteristic curves
TCGA: the cancer genome atlas.
